# CSCdb: a cancer stem cells portal for markers, related genes and functional information

**DOI:** 10.1093/database/baw023

**Published:** 2016-03-17

**Authors:** Yi Shen, Heming Yao, Ao Li, Minghui Wang

**Affiliations:** 1 School of Information Science and Technology; 2 School of Life Science; 3 Centers for Biomedical Engineering, University of Science and Technology of China, Hefei, AH230027, China

## Abstract

Cancer stem cells (CSCs), which have the ability to self-renew and differentiate into various tumor cell types, are a special class of tumor cells. Characterizing the genes involved in CSCs regulation is fundamental to understand the mechanisms underlying the biological process and develop treatment methods for tumor therapy. Recently, much effort has been expended in the study of CSCs and a large amount of data has been generated. However, to the best of our knowledge, database dedicated to CSCs is not available until now. We have thus developed a CSCs database (CSCdb), which includes marker genes, CSCs-related genes/microRNAs and functional annotations. The information in the CSCdb was manual collected from about 13 000 articles. The CSCdb provides detailed information of 1769 genes that have been reported to participate in the functional regulation of CSCs and 74 marker genes that can be used for identification or isolation of CSCs. The CSCdb also provides 9475 annotations about 13 CSCs-related functions, such as oncogenesis, radio resistance, tumorigenesis, differentiation, etc. Annotations of the identified genes, which include protein function description, post-transcription modification information, related literature, Gene Ontology (GO), protein-protein interaction (PPI) information and regulatory relationships, are integrated into the CSCdb to help users get information more easily. CSCdb provides a comprehensive resource for CSCs research work, which would assist in finding new CSCs-related genes and would be a useful tool for biologists.

**Database URL:** http://bioinformatics.ustc.edu.cn/cscdb

## Introduction

Cancer stem cells (CSCs), which have the ability to self-renew and to differentiate into various tumor cell types, are a special class of tumor cells (1). As CSCs are resistant to chemotherapy and radiotherapy and have a strong tumorigenic potential, conventional treatment strategy cannot eliminate CSCs thoroughly and often lead to the recurrence (2). CSCs have aroused widespread concern and more and more articles about CSCs have been published (3).

Identifying CSCs-associated genes and their functional information is one of the central tasks in CSCs research work. Identifying and isolating CSCs are the first stage of the research work and are also the basis of the further experiments (4). Marker genes are usually utilized to label the CSCs or to distinguish CSCs from common cancer cells (5). With the help of marker genes, the difficulty of CSCs identification and isolation has been reduced greatly (3). Researchers also find many CSCs-related genes, which can influence the cellular regulation in CSCs. Some of these genes have been proved to be responsible for the drug resistance and many other genes may be associated with the tumor recurrence (2). All this information is critical for finding new cancer treatment strategy and is valuable for mechanism research. However, as far as we known, such information is scattered in a large number of literature, which makes researchers difficult to obtain useful information efficiently.

To date, many cancer-related databases have been built. These databases provide cancer-related information and are valuable tools for tumor research work. For example, CaGe (http://mgrc.kribb.re.kr/cage/pageHome.php?m=hm) is a cancer gene annotation server, which affords functional annotations of cancer-related genes. GeneCards (6) is a wildly used database that contains comprehensive functional information of human genes. There are also many databases providing biomarkers of cancer cells, such as CacerDriver (http://www.cancerdriver.com) and Brain Tumor Medical Database (http://www.brainlife.org/database.htm). All these databases are useful for cancer-related studies. However, in these databases, information regarding the CSCs, such as marker genes, CSCs-related genes and their functional annotations, are not covered. To the best of our knowledge, few databases are focuses on the CSCs. Therefore, we developed the database, CSCs database (CSCdb), to fill this gap and to capture the intrinsic features of CSCs-related genes.

CSCdb currently contains 74 marker genes of  > 25 tissues, 1769 CSCs-related genes and 9475 functional annotations. All these data were gathered from literature manually and have been carefully reviewed. CSCdb provides the information of CSCs-related genes such as gene keywords, GO terms and functional annotations. Our database also integrated gene annotations from other public databases to help users to obtain comprehensive information more easily. In the CSCdb, users can find the reported marker genes easily and get the gene functional annotations quickly. The website is designed to provide user-friendly access and assist users in the CSCs research work.

## Materials and methods

### Data types, literature search and data collection

We manually collected known CSCs marker genes and functional annotations from the published literature. All the literature was downloaded from two databases: PubMed database and Web of Knowledge. To collect CSCs-related articles, we performed a query of PubMed by using the keywords ‘cancer stem cell’, ‘tumor stem cell’, ‘carcinoma stem cell’ or ‘tumor initiating cell’. The same keywords were used in the query of Web of Knowledge. After removing duplicate articles presented in both databases and the articles without abstract, about 13 000 articles were used for information extraction and data collection. The process of extracting useful information from literature included three steps. First, we removed the extraneous records based on the topics of the abstracts. Then, we read the articles and extracted descriptions of marker genes, CSCs-related genes and functional annotations. The definition of ‘related’ mainly includes function related and expression related. A gene is ‘function related’ with CSCs means that (i) this gene can regulate cellular process in CSCs or take part in the process; (ii) influence the CSCs properties, such as chemo-resistance, radio-resistance; (iii) indicate the outcome of therapy. The ‘expression related’ means that a gene might be differentially expressed between CSCs and common cancer cells or high-expressed/low-expressed in a sub-group of CSCs that have a different properties, such as CSCs with stronger carcinogenicity. After that, we carefully mapped the gene names to Entrez IDs. Furthermore, the mapping results were checked by two people manually. In CSCdb, marker genes include both cell surface proteins and intracellular proteins.

### Gene annotations

In order to make users get comprehensive information of genes in CSCdb more easily, we also integrated gene annotations from public databases. Basic gene information, such as recommend name, chromosome location and gene description, was extracted from NCBI (7) and Uniprot (8). MicroRNA and transcription factor regulation relationships were downloaded from MirTarbase (9) and AnimalTFDB (10). The protein–protein interactions provided by STRING database were also integrated (11). Additionally, as most marker genes and CSCs-related genes are protein-coding genes, we also collected the post-translational modifications (PTM) sites of different PTM types from dbPTM (12). PTM types included acetylation, methylation, sumoylation, ubiquitylation and phosphorylation. The gene ontology information, which is shown in a table, can also be found in the ‘gene page’. For each gene in CSCdb, we also provided the pathways that it takes part in. All the pathway information is provided by Reactome (13) and is displayed with a table.

### Functional annotations

All the gene functional annotations in CSCdb were extracted from published literature manually. The functional annotations in CSCdb mainly include 13 categories, which are angiogenesis, apoptosis, carcinogenesis, chemoresistance, differentiation, eradication, malignancy, metastasis, prognosis, radioresistance, recurrence, self-renewal and telomerase. These functional annotations, valuable for CSCs researchers, are most frequently appeared in the published CSCs-related articles. Not only the protein-coding genes, we also collected the functional annotations of many microRNAs. Each annotation includes reference link, name of gene/microRNA and the reference text.

### Enrichment analysis

To find out the biological pathways that the CSCs-related genes are involved in, we performed pathway enrichment analysis by DAVID (14), which is a wildly used bioinformatics tool for gene ID mapping and gene functional analysis. In the enrichment analyses, all human genes were used as background and the program was run with default parameters. For the total CSCs-related genes pathway enrichment analysis, all CSCs-related genes were used as the DAVID input and the results can be downloaded from the ‘Download’ page. We also performed the pathway enrichment analysis for 13 gene categories. Take self-renewal for example, we first got all genes related to self-renewal from the CSCdb and then used these genes as the input of DAVID server to find pathways, in which these self-renewal related genes are enriched. The statistically significant pathways (*P*  <  0.01) can be downloaded from the ‘Statistics’ page

### Network visualization

In CSCdb, Cytoscape Web plugin (15) was used for regulation network and PPI network visualization. Cytoscape Web plugin is a wildly used open source javascript library that can be used to render interactive networks in popular used web browsers. The regulatory network and PPI network were stored in MySQL database and displayed with Cytoscape Web plugin.

### Finding similar gene

In CSCdb, we used GS2 (16) to measure the functional relatedness of a gene set, which provides functional distances of each gene pair. GS2 quantifies the similarity between two different genes based on the GO annotation of these genes and can provide comparable similarity scores to other established methods. In CSCdb, we used GS2 to quantify the similarity between different genes and provided 20 most similar genes for each gene.

#### Database architecture and web interface

CSCdb is an instance of MySQL (version 5.541), a relational database that worked at the backend to store the collected data. All information collected from literature was converted to structured data stored in tables. Related fields in tables were linked as foreign keys and were indexed for fast information retrieval. HTML/CSS/JavaScript were used for building a user-friendly web interface and all web pages were implemented in PHP language. Other software technologies, such as JQueryUI and Cytoscape Web, were used for data presentation. The gene information and annotations are dynamically retrieved from the database using SQL and will keep updating.

## Results

CSCdb is designed to be a user-friendly database, providing the information of CSCs. CSCdb is freely accessible and no password or registration is required. A brief description of CSCs and CSCdb can be found in the ‘home page’. In the ‘home page’, there is also an introduction to the data resources, which would help users find the needed information. All web pages contain a top menu, which includes ‘browse’, ‘download’, ‘help’ and so on. This can help users find the needed function quickly. Users, who want to ask questions or provide a feedback can find the Email address in the ‘contact us page’. We will continuously update the database and provide a useful resource to facilitate the CSCs research work.

### Database summary

**Figure 1.**
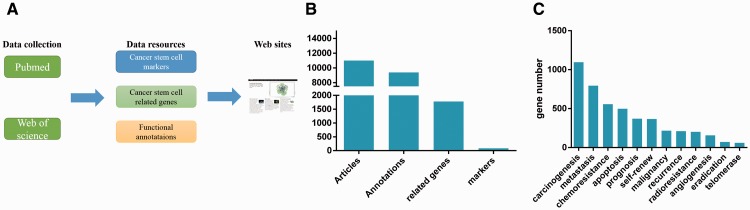
Database overview. (**A**) Summary of the database, which shows the resources types and source of the literatures. (**B**) As showed in the figure, there are 74 marker genes, 1769 related genes and 9475 functional annotations. The functional annotations of marker genes and CSCs-related genes are mainly about 13 categories. (**C**) The gene number involved in different function is very different.

The database contains four types of resource: (i) CSCs that have been identified, (ii) marker genes, (iii) CSCs-related genes/microRNAs, (iv) functional annotations of marker genes or CSCs-related genes/microRNAs (Figure 1A). In our database, we collected the marker genes of  > 25 tissues. The CSCdb includes 74 marker genes, 1769 CSCs-related genes/microRNAs, and 9475 functional annotations obtained from about 13 000 articles (Figure 1B). Each annotation includes at least one gene/microRNA (marker gene, CSCs-related gene or CSCs-related microRNA). As mentioned above, the functional annotations include 13 categories. The gene number involved in different functions varied considerably, from less than one hundred genes to more than one thousand genes (Figure 1C).

### Web interface

**Figure 2.**
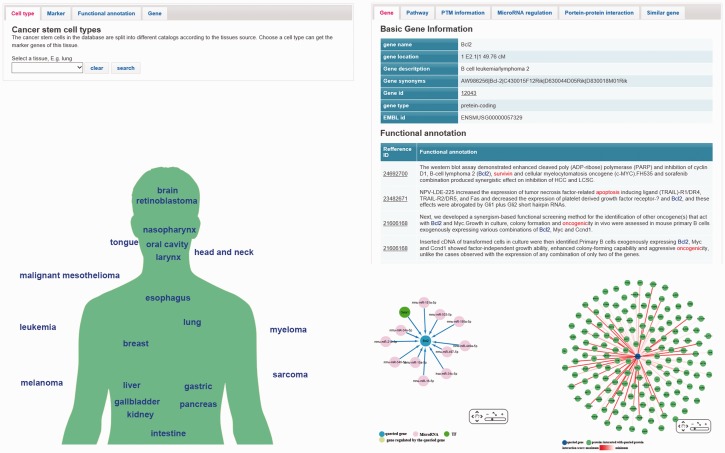
The web interface of the database. (**A**) The ‘brows page’ contains four tabs include cell type, markers, function and related gene. Users can choose different tabs and access the content of the database. (**B**) Users can also choose a marker category in the ‘marker map page’. (**C**) The ‘gene page’ shows the information of a gene. Interactive network visualization of PPI and microRNA-gene interaction are created by Cytoscape Web plugin (a JavaScript library).

The ‘browse page’ contains four tabs (Figure 2A), which are designed for different purposes. In each tab, there is a brief introduction. Users can browse the database by clicking ‘tissue type’, ‘functional annotation’, ‘marker’ and ‘related genes’. In the ‘tissue type’ tab, users can browse marker genes of a specific tissue. Clicking the maker name will link to the ‘gene page’ that provides details of this marker. An alternative way of browsing marker genes is using ‘marker map page’ (Figure 2B), which lists the tissues on a schematic diagram of human body. In ‘functional annotation’ tab, users can choose an interested function and find the genes that involved in regulation of this function. These genes include marker genes and CSCs-related genes which are displayed with two different tables. In ‘marker’ and ‘related gene’ tabs, users can get the list of all markers or CSCs-related genes/microRNAs. Users can also search a gene by using the ‘Search’ page. Details of a marker or a CSCs-related gene are shown in the ‘gene page’ (Figure 2C), which includes following information: (i) Basic gene information such as, gene ids, key words, GO terms, chromosome position, PTM sites and pathway information; (ii) Marker records and functional annotations. Both marker records and functional annotations provide reference ids that can link to the reference articles; (iii) PPI information and regulatory information. Both the PPI and regulatory information are illustrated with interactive networks using the Cytoscape Web plugin. The regulatory information includes microRNA regulation and transcription factor regulation. The experiment evidences of each regulation relationship are also available; (iv) Similar genes. In the ‘gene page’, users can also find 20 genes that are most similar with the queried gene. Each similar gene has a functional distance score provided by GS2. This information may help researchers find new cancer-related genes or discovery new function of a known CSCs-related genes.

### Enrichment analysis

To investigate the CSCs-related genes involved pathways, we performed the KEGG pathway enrichment analysis. The results show that in the top 25 pathways, 14 pathways are tumor specific, such as ‘Pathways in prostate cancer’, ‘Pathways in pancreatic cancer’, ‘Pathways in bladder cancer’ (Table 1). Additionally, a large number of genes enriched in cancer-related pathways, including ‘p53 signaling pathway’, ‘Cytokine-cytokine receptor interaction pathway’, ‘ErbB signaling pathway’, ‘MAPK signaling pathway’ and ‘mTOR signaling pathway’. All these pathways are associated with the cancer process (17–19) and play an important role in the pathogenesis of tumor. The CSCs-related genes are also enriched in some cellular regulation processes such as autophagy and cell cycle regulation. The enrichment results can be downloaded from the ‘Downloaded’ page.

**Table 1.** Pathway enrichment analysis of CSCs-related genes (top 25)

Pathway term	*P* value	FDR
Pathways in cancer	4.83E−49	5.95E−46
Pathways in prostate cancer	1.26E−23	1.55E−20
Cytokine-cytokine receptor interaction	7.12E−18	8.78E−15
Pathways in pancreatic cancer	1.00E−17	1.24E−14
Pathways in chronic myeloid leukemia	7.85E−17	1.33E−13
p53 signaling pathway	2.24E−14	2.76E−11
Pathways in bladder cancer	2.72E−14	3.35E−11
Pathways in colorectal cancer	6.47E−13	7.98E−10
Pathways in melanoma	8.72E−13	1.08E−09
Hematopoietic cell lineage	8.73E−12	1.08E−08
Pathways in glioma	1.57E−11	1.94E−08
Pathways in renal cell carcinoma	1.81E−11	2.24E−08
Focal adhesion	4.61E−11	5.69E−08
ErbB signaling pathway	6.56E−11	8.08E−08
Cell cycle	1.98E−10	2.44E−07
Pathways in acute myeloid leukemia	2.36E−10	2.91E−07
Pathways in endometrial cancer	3.30E−10	4.06E−07
Jak-STAT signaling pathway	7.99E−10	9.85E−07
Apoptosis	1.32E−09	1.62E−06
Neurotrophin signaling pathway	6.32E−09	7.79E−06
Pathways in small cell lung cancer	7.57E−09	9.34E−06
Toll-like receptor signaling pathway	1.11E−08	1.37E−05
MAPK signaling pathway	4.07E−08	5.01E−05
Pathways in non-small cell lung cancer	1.54E−07	1.90E−04
mTOR signaling pathway	3.13E−07	3.86E−04

## Download

All the datasets in CSCdb can be downloaded freely. Users can explore the ‘download page’ and download the needed dataset following the file description. Users can right click the download link and save the datasets to their own computer. All the datasets are saved in Excel format with a short description of each column.

## Discussion

CSCdb is a literature-based database for the CSCs research, which integrated marker genes, CSCs-related genes and functional annotations. It might be a valuable resource for the identification of new CSCs and finding potential therapeutic targets. It can be also helpful in developing bioinformatics tools to find new CSCs-related genes. All the date in the database is freely available for downloading and further analysis.

With the data in CSCdb, we made a comparisons among ‘CSCs-related genes’, ‘cancer genes’ and ‘reprogramming and stem cell-related genes’ by comparing the pathways, GO-terms and InterPro domains that these three types of genes are enriched in. The ‘cancer genes’ were downloaded from Cosimic (20) and Tsgene (21) while ‘reprogramming and stem cell-related genes’ were provided by RPdb (22). We performed the pathway enrichment analysis using the DAVID server. For each type of genes, we selected top 25 pathways for comparison. As shown in Figure 1S (A), all these three types of genes are enriched in 16 pathways, including Jak-STAT pathway, MAPK pathway and ErbB pathway. In the top 25 pathways of CSCs-related genes, 20 pathways are also included in the top 25 pathways of ‘cancer genes’. The same comparisons were performed using GO terms and InterPro domains and the results were shown in Figure 1S (B, C). ‘CSCs-related genes’ share 12 GO terms and 8 InterPro domains with both ‘cancer genes’ and ‘reprogramming and stem cell-related genes’. We also find that many stemness genes, such as OCT-4, SOX2 and NANOG, are all participated in the cellular regulation in CSCs.

In the coming years, we will continue optimize the database structure and the user interface. Additional markers and related genes will be added to the database if new information is available and the database will keep updating regularly. We expect the CSCdb will become valuable resource for CSCs researchers and facilitate the new CSCs-related genes identification.

## Supplementary Material

Supplementary Data
