# Supplementary material for: CSCdb: a cancer stem cells portal for markers, related genes and functional information
Source: Database (Oxford). 2016 Mar 17;2016:baw023. doi: 10.1093/database/baw023 (PMC4795926; doi:10.1093/database/baw023)
Supplement: Supplementary Data [file supp_2016_baw023_index.html]

Supplementary Data 

# CSCdb: a cancer stem cells portal for markers, related genes and functional information

## Supplementary Data

files

- Supplementary Data - docx file
