# Supplementary material for: CSCdb: a cancer stem cells portal for markers, related genes and functional information
Source: Database (Oxford). 2016 Mar 17;2016:baw023. doi: 10.1093/database/baw023 (PMC4795926; doi:10.1093/database/baw023)
Supplement: Supplementary Data [file supp_baw023_supplementary.docx]

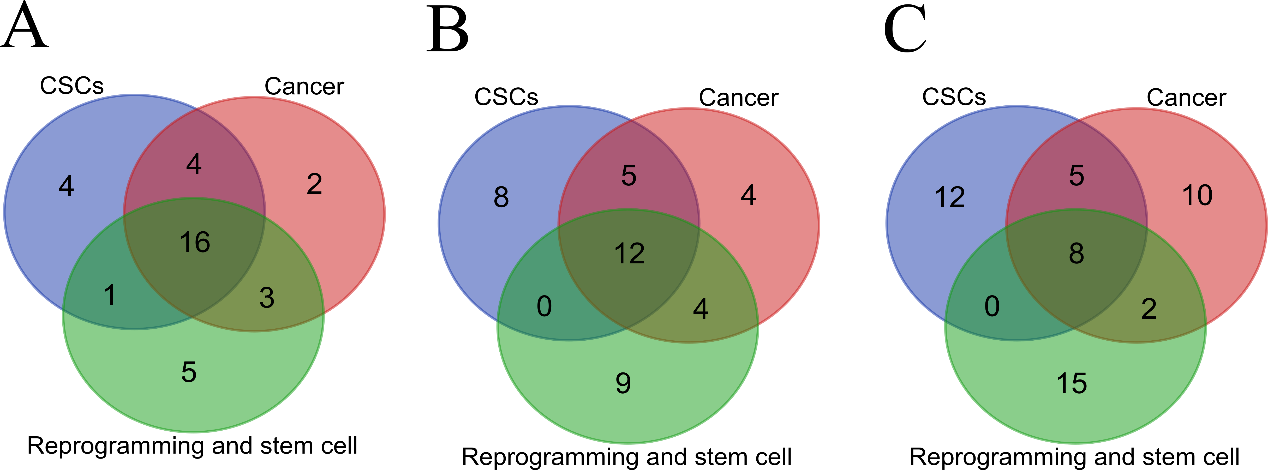


**Fig 1S. Comparisons among “CSCs related genes”, cancer genes and “Reprogramming and stem cell” related genes**

A. Comparisons of enriched pathways. Top 25 pathways of each gene type were selected for the comparisons. B. Comparisons of enriched Go terms. C. Comparisons of enriched InterPro domain.
